# Identification and characterization of thirty novel microsatellite DNA markers from the Chinese mitten crab *Eriocheirsinensis* expressed sequence tags

**DOI:** 10.1186/s13104-016-1909-6

**Published:** 2016-02-17

**Authors:** Jingjing Li, Xuyun Geng, Limei Chen, Jinsheng Sun

**Affiliations:** Tianjin Key Laboratory for Animal and Plant Resistance, College of Life Sciences, Tianjin Normal University, 393 Binshuixi Rd, Xiqing District, Tianjin, 300387 China; Tianjin Diseases Prevention and Control Center of Aquatic Animals, 442 Jiefangnan Rd, Hexi District, Tianjin, 300221 China; Tianjin Key Laboratory of Aqua-Ecology and Aquaculture, Fisheries Science Department, Tianjin Agricultural University, 22 Jinjing Rd, Xiqing District, Tianjin, 300384 China

**Keywords:** Microsatellites, EST, Chinese mitten crab, *Eriocheir sinensis*

## Abstract

**Background:**

The Chinese mitten crab *Eriocheir sinensis* is an economically important decapod crustacean in China. Despite a widespread distribution and production in China, the resources of *E. sinensis* have experienced a dramatic decline in the past decades. Here we describe a new set of novel polymorphic microsatellite loci to facilitate the investigation of genetic structure and artificial breeding.

**Results:**

In this study, a set of 30 novel polymorphic microsatellite markers for *E. sinensis* was developed from EST databases. The number of alleles per locus ranged from three to twenty. The observed and expected heterozygosities ranged from 0.047 to 0.932 and from 0.047 to 0.935, respectively.

**Conclusions:**

These informative microsatellite markers will be useful in studies of genetics, genomics and marker-assisted selection breeding in *E. sinensis*.

## Findings

### Background

The Chinese mitten crab *Eriocheir sinensis* is one of the most economically important aquaculture species in China [1] due to its taste and nutritious value, with a native range extending from the coastal estuaries of Korea in the north to the Fujian province of China in the south. However, the wild populations of *E. sinensis* have experienced a dramatic decline in the past decades due to overfishing and water pollution [2]. In China, the basic production technology of mitten crab populations has had a long history, with the conventional selective breeding programs based on phenotypic assessment. At present, the yield of *E. sinensis* is almost completely from artificial breeding. Unfortunately, like many other cultured species, the aquaculture performance of *E. sinensis* has declined significantly. In order to protect genetic diversity and prevent population degradation, understanding population genetic structure and genetic connectivity among populations and making a genetic linkage map are necessary.

Microsatellite markers provide a powerful tool in genome researches due to their wide distribution, codominant inheritance and high polymorphism. To date, approximately 83 microsatellite markers have been developed and applied for *E. sinensis* [3–8]. Although the number of described loci is relatively high, much more works is still needed because of the large diploid chromosome number of *E. sinensis* (2*n* = 146) [9]. In this study, we describe a new set of 30 EST-derived microsatellite markers which would aid in characterizing population structure, genetic diversity and constructing linkage map in *E. sinensis*.

### Experimental section

A total of 17067 *E. sinensis* ESTs obtained from the GenBank database (2013) were screened using SSRIT program [10] that was designed to find regions containing microsatellites. The parameters were set for detection of di-, tri- and tetranuclotide motifs with a minimum of six repeats, respectively. Eighty-five microsatellite loci were selected for microsatellite marker optimization. Primers flanking microsatellite were designed using the PRIMER PREMIER 5.0 program.

Sixty cultured *E. sinensis* individuals were randomly captured from Xieyuan Fishing Company in Qilihai region in Tianjin City, China. Genomic DNA was extracted from the leg muscles using a modified phenol-chloroform protocol [11]. Polymerase chain reaction (PCR) amplifications were performed in 10-μL volumes containing 0.25 U *Taq* DNA polymerase (Takara), 1× PCR buffer, 0.2 mM dNTP mix, 1 *μ*M of each primer set, 1.5 mM MgCl_2_ and about 100 ng template DNA. The PCR profiles for all loci were an initial denaturing at 94 °C for 3 min, followed by 35 cycles of 1 min at 94 °C, 1 min at the annealing temperatures listed in Table 1, and 1 min at 72 °C, with a final extension step of 5 min at 72 °C on a MJ Research PTC-200 DNA Engine (Peltier Thermal Cycler). Amplification products were resolved via 6 % denaturing polyacrylamide gel, and visualized by silver-staining. A 10-bp DNA ladder (Invitrogen) was used as a reference marker for allele size determination. The calculations of observed and expected heterozygosities were estimated with the program MICROSATELLITE ANALYSER software [12]. Tests for linkage disequilibrium and deviations from Hardy–Weinberg equilibrium (HWE) were performed using GENEPOP 4.2 [13, 14].

**Table 1:** Characterization of 30 EST-SSRs in the Chinese mitten crab *Eriocheir sinensis*

Locus	GenBank accession no.	Repeat motif	Primer sequence (5′–3′)	*T*_a_ (°C)	No. of individuals	No. of alleles	Size range (bp)	*H*_O_	*H*_E_	*P* value
ES27	FL571941	(GT)_11_	F: TGTGATGAGAAGAAACCAAAGA	52	59	8	107–123	0.712	0.797	0.0005*
			R: AATACCTGCTGGCGATGA							
ES39	FG359711	(GT)_17_	F: AGGACGAAAGTTGGAGGG	55	56	8	114–128	0.482	0.864	0.0000*
			R: AAATACAAATCTACGGGAGACAC							
ES104	FL569100	(TG)_21_	F: TCACAACTACGAAAACCT	48	53	3	190–200	0.047	0.047	1.0000
			R: GAGTGTCAGTGTATGGAAT							
ES108	FG984086	(GT)_19_	F: GTAAACCCTACGAAACCATA	54	59	15	91–119	0.932	0.932	0.0000*
			R: ACTCCCTAAACTACCTAACTACCA							
ES130	FL570152	(GGC)_7_	F: CGTTCGTTGTGAGCGTCTGC	57	60	4	145–154	0.167	0.159	1.0000
			R: CGCCTGGTCCATCTCATCG							
ES212	FG984116	(CCA)_6_(CAA)_10_	F: GTGACACTGATGCCTGACGA	55	56	4	181–190	0.482	0.583	0.2114
			R: TTATGCCTTTATTGACCGAGAC							
ES271	FG359821	(TG)_12_	F: GCTTCTCACCCGTGATGT	54	49	6	176–186	0.615	0.754	0.0065
			R: CTCCTCCTTTGCTTTCTTTA							
ES352	FL572494	(GT)_10_	F: CACTCGGTACAAACATCAC	53	56	17	91–127	0.768	0.929	0.0000*
			R: AATGGGTATGGATTTAGTGT							
ES582	FL570362	(GA)_26_	F: ACCTCCAAGCCCCTTACC	53	58	5	241–261	0.293	0.296	0.6958
			R: GAACAAACACGAGGGACAAC							
ES584	FL574606	(CA)_22_	F: AGGGAAGTTGTAAAGGTAAGGA	49	56	9	222–240	0.429	0.863	0.0000*
			R: ATGGGAATGAGATGAGGATAGA							
ES645	FL571837	(AC)_13_	F: GACGCACGACAACAACCTC	60	56	11	122–158	0.522	0.912	0.0000*
			R: CCACTCCTAGTCAACGGAAAGA							
ES709	FL574505	(GCA)_7_	F: GCAGCCACAACCAGCAGAAG	60	60	6	197–212	0.233	0.219	1.0000
			R: CTCGCCATGCAGGATCACC							
ES776	FG357327	(CA)_34_	F: GTTGGTGTTGAAGGAGCCA	53	59	4	204–220	0.410	0.557	0.1180
			R: CTTAATCCGTTGCGTCAGC							
ES789	GE340666	(GT)_25_	F: TCGGGTGAGTTAGGTGTAGG	52	53	18	178–218	0.170	0.929	0.0000*
			R: AGCAAGGCACTTTGAAGC							
ES851	GE340258	(CAC)_7_	F: TCCAACCAGGCGGCAAAG	54	54	7	216–240	0.778	0.814	0.0580
			R: AGCAAGTCCACCGAACACCAT							
ES911	FL569216	(TTCA)_7_	F: CGGCGAGACTCACGAACT	56	53	5	242–258	0.453	0.634	0.0012*
			R: CGAGGGTGAAGAGGCATT							
ES998	FL572952	(GT)_5_N(TG)_5_N_2_(TG)_12_	F: CGACGGTGTCAGATTAGTG	56	51	8	217–231	0.392	0.860	0.0000*
			R: ACCAACGGGCTCAAGAAG							
ES1045	FL575077	(GT)_28_	F: GGAGCACCACCGTAAAGATA	55	55	17	112–148	0.582	0.935	0.2415
			R: TCAACACGAAACCGCCAC							
ES1053	FL572054	(CA)_10_	F: CTACACCAAGACCTCCTCGT	57	58	6	145–155	0.741	0.703	0.3476
			R: GGCTGGTTTGTTGGGTAAG							
ES1126	FG359126	(AAGG)_12_	F: TGTCCAGTCTCCCATCAA	54	60	14	180–240	0.883	0.907	0.7140
			R: TGGTATGGTCGCTAATCTC							
ES1139	FL569992	(GA)_11_	F: ACAGACGCACCTCCAAGC	55	59	20	110–150	0.644	0.925	0.0000*
			R: TTAGAACAAACACGAGGGACA							
ES1171	FG357452	(AC)_8_N_5_(TC)_6_	F:CAATCTGCCCTAATCTGTCTGTAA	57	55	8	156–172	0.900	0.871	0.0000*
			R:GGGAAAGGTAGGAGGATAAGTGA							
ES1178	GE341515	(ACC)_7_	F: TCCCATCGCCGTAGAAAC	55	60	3	138–146	0.150	0.144	1.0000
			R: ACGCCAGACTGGACAAGC							
ES1240	FG982578	(TACA)_11_	F: ATTGTAGCCATACCAGCAT	52	60	13	152–200	0.883	0.890	0.3087
			R: ACAAATCTTACAACTACGGC							
ES1289	FL574500	(TTA)_13_	F: ACCTTGTGGATACCAGCAT	50	55	12	124–169	0.782	0.872	0.0000*
			R: TTCCCTTCAACCATACATAA							
ES1293	FL569028	(GTA)_7_N_8_(GTA)_5_	F: GCCTCAATATCGGGCTTAT	55	59	7	129–176	0.763	0.769	0.2826
			R: CCTCCCTGCGACTTCTACT							
ES1300	FL575122	(TG)_10_	F: CCCTTGTTGATTGCCCTA	55	52	5	186–194	0.519	0.608	0.0846
			R: GTCACGAAGAAGCACCTC							
ES1482	FL576108	(TG)_7_	F: ACTATCCCTGCCTCACTACCG	57	60	7	115–129	0.250	0.522	0.0000*
			R: GAACAAACATTACCGTCACTCG							
ES1507	FL572842	(AGT)_5_N_2_(TAG)_9_	F: TGGAGTAGGTCGGTTCGGT	57	58	6	197–215	0.603	0.743	0.0299
			R: TAGCAACATCCCTCGTCCTC							
ES1513	FL574610	(AG)_9_	F: AACAGTGGCAGGAAACAGAAAG	57	56	7	100–114	0.217	0.739	0.0000*
			R: AGGGAAGGATGAGTGTGAGCA							

*T*_a_ annealing temperature, *H*_O_ observed heterozygosity, *H*_E_ expected heterozygosity

* Significant departure (*P* < 0.05) from expected Hardy–Weinberg equilibrium conditions after correction for multiple tests (*k* = 30)

### Results and discussion

Of the 85 potential microsatellite markers, forty-four loci were successfully amplified with the expected products. Thirty of them revealed polymorphism among the tested 60 individuals of *E. sinensis*. The number of alleles at each locus ranged from three to twenty with an average of 8.767 alleles per locus (Table 1). Observed heterozygosities ranged from 0.047 to 0.932 with an average of 0.527, while expected heterozygosities ranged from 0.047 to 0.935 with an average of 0.693. The mean number of alleles per locus, *H*_O_ and *H*_E_ demonstrated a relatively high genetic diversity within crab individuals. This was similar to reports from studies in other locations [3, 4, 6, 8]. Fourteen of the 30 loci significantly deviated from the Hardy–Weinberg equilibrium after Bonferroni correction. This might be due to the limited sample size, and/or the presence of null alleles at these loci. The high polymorphism of the loci suggests that they would be useful tool in studies of population structure, genetic diversity and the construction of genetic map for *E. sinensis*.

### Conclusions

A set of 30 novel hypervariable microsatellite loci in *E. sinensis* was reported in this study. All the characterized microsatellite markers are suited for assessing the genetic diversity and the population structure, and also facilitate marker-assisted selection breeding of *E. sinensis*.

### Ethics statement

Every effort was made to minimize animal pain, suffering and distress and to reduce the number of animal used. Sampling of the crabs was approved by Tianjin Diseases Prevention and Control Center of Aquatic Animals.

### Availability of the supporting data

The microsatellite sequences are available through the National Centre for Biotechnology Information (http://www.ncbi.nlm.nih.gov); GenBank accession numbers see Table 1.
